# Why is Tanimoto index an appropriate choice for fingerprint-based similarity calculations?

**DOI:** 10.1186/s13321-015-0069-3

**Published:** 2015-05-20

**Authors:** Dávid Bajusz, Anita Rácz, Károly Héberger

**Affiliations:** Medicinal Chemistry Research Group, Research Centre for Natural Sciences, Hungarian Academy of Sciences, Magyar tudósok körútja 2, H-1117 Budapest XI, Hungary; Plasma Chemistry Research Group, Research Centre for Natural Sciences, Hungarian Academy of Sciences, Magyar tudósok körútja 2, H-1117 Budapest XI, Hungary; Department of Applied Chemistry, Faculty of Food Science, Corvinus University of Budapest, Villányi út 29-43, H-1118 Budapest XI, Hungary

**Keywords:** Fingerprint, Similarity, Ranking, Data fusion, Analysis of variance, Sum of ranking differences, Distance metrics

## Abstract

**Background:**

Cheminformaticians are equipped with a very rich toolbox when carrying out molecular similarity calculations. A large number of molecular representations exist, and there are several methods (similarity and distance metrics) to quantify the similarity of molecular representations. In this work, eight well-known similarity/distance metrics are compared on a large dataset of molecular fingerprints with sum of ranking differences (SRD) and ANOVA analysis. The effects of molecular size, selection methods and data pretreatment methods on the outcome of the comparison are also assessed.

**Results:**

A supplier database (https://mcule.com/) was used as the source of compounds for the similarity calculations in this study. A large number of datasets, each consisting of one hundred compounds, were compiled, molecular fingerprints were generated and similarity values between a randomly chosen reference compound and the rest were calculated for each dataset. Similarity metrics were compared based on their ranking of the compounds within one experiment (one dataset) using sum of ranking differences (SRD), while the results of the entire set of experiments were summarized on box and whisker plots. Finally, the effects of various factors (data pretreatment, molecule size, selection method) were evaluated with analysis of variance (ANOVA).

**Conclusions:**

This study complements previous efforts to examine and rank various metrics for molecular similarity calculations. Here, however, an entirely general approach was taken to neglect any *a priori* knowledge on the compounds involved, as well as any bias introduced by examining only one or a few specific scenarios. The Tanimoto index, Dice index, Cosine coefficient and Soergel distance were identified to be the best (and in some sense equivalent) metrics for similarity calculations, *i.e*. these metrics could produce the rankings closest to the composite (average) ranking of the eight metrics. The similarity metrics derived from Euclidean and Manhattan distances are not recommended on their own, although their variability and diversity from other similarity metrics might be advantageous in certain cases (*e.g.* for data fusion). Conclusions are also drawn regarding the effects of molecule size, selection method and data pretreatment on the ranking behavior of the studied metrics.

**Graphical Abstract:**
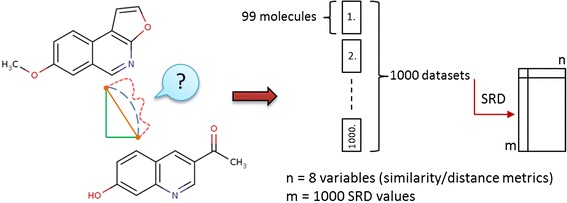
A visual summary of the comparison of similarity metrics with sum of ranking differences (SRD).

**Electronic supplementary material:**

The online version of this article (doi:10.1186/s13321-015-0069-3) contains supplementary material, which is available to authorized users.

## Background

Quantifying the similarity of two molecules is a key concept and a routine task in cheminformatics [1-3]. Its applications encompass a number of fields, mostly medicinal chemistry-related, such as virtual screening [4]. Although some commonly applied best practices for molecular similarity calculations exist, they are mostly based on practical experience. Meanwhile, a virtually infinite “method space” is available and waiting to be explored, with a plethora of molecular representations and a significant number of similarity (or conversely, distance) definitions to compare these representations. Even though much effort has been made to reveal and assess numerous possibilities, our knowledge is still relatively scarce about the effects the choice of methods has on the outcome of molecular similarity calculations and rankings.

Previous work aiming to compare and assess such methods includes a 2009 article by Bender and coworkers, in which 37 molecular fingerprints were compared and *their* similarities were quantified (based on their rank-orderings of the same dataset) by means of statistical methods, such as principal component analysis (PCA) [5]. They were able to estimate the extent to which the information captured by these descriptors overlap, and also to visualize them in a three-dimensional space. Despite the fact that diverse fingerprints (*i.e*. fingerprints that capture different aspects of molecular structure) could be identified, the use of multiple fingerprints for consensus scoring only marginally improved the results obtained with a single fingerprint. However, using different fingerprints, different (active) molecules were retrieved, which suggests the use of orthogonal fingerprints individually in virtual screenings. Based on their evaluation with the calculation of retrieval rates of active molecules, extended connectivity fingerprints performed best (although only slightly better from the runner-up SEFP4, LCFP4 and FCFP4/6 fingerprints), regardless of diameter (*i.e.* ECFP4 and ECFP6 performed equally well, the notations are explained in the corresponding reference) [6].

In a 2014 paper Cereto-Massagué and coworkers conclude that most of the commonly used and popular fingerprints have very similar performances, inter-target differences for the same fingerprint being usually greater than the differences for different fingerprints for the same target molecule [7]. They also conclude that under the same conditions, circular fingerprints usually perform best.

Similarity (or distance) metrics are employed in a wide variety of areas, stimulating the assessment of their performance in *e.g.* texture image retrieval [8], webpage clustering [9] or event identification in social media [10]. From an area that is more closely related to cheminformatics, a 2013 article by Reisen and coworkers compares 16 similarity measures based on their performances in high-content screening (HCS) [11]. They conclude that nonlinear correlation-based similarity metrics such as Kendall’s τ and Spearman’s ρ outperformed other frequently used metrics, such as the Euclidean distance (for HCS).

Several studies have also been published on the comparison of similarity metrics in cheminformatics-related fields, mostly by Peter Willett’s group at the University of Sheffield. In a 2002 article, they compare 22 similarity metrics [12]. In their conclusions, they reinforce the popularity of the Tanimoto coefficient and they suggest several other similarity metrics for data fusion. In the same year, Chen and Reynolds suggest the use of the Tanimoto index instead of the Euclidean distance for 2D Fragment-Based Similarity Searching [13]. A year later Salim and coworkers find that combinations of 2–4 similarity metrics can outperform the Tanimoto index, although no combination shows consistently high performance across different scenarios [14]. In a 2006 review, Willett maintains, among other conclusions that “the well-established Tanimoto is the coefficient of choice for computing molecular similarities unless there is specific information about the sizes of the molecules” [15].

In a 2013 article Todeschini and coworkers perform the comparison of 51 similarity coefficients, their conclusions also support the usefulness of the Tanimoto index, as well as identifying two additional metrics “that may be worthy of future study for applications in chemoinformatics” [16]. Willett’s group has also extensively studied possible applications of data fusion techniques to improve the performance of similarity calculations [17]. He reported that data fusion was able to enhance the performance of similarity-based virtual screening in two different approaches as well: similarity fusion (where more similarity measures are used with a single reference structure) and group fusion (where a single similarity measure is used with more reference structures), concluding however that “group fusion is generally far superior to similarity fusion”. In an earlier work, they identified the Tanimoto coefficient as the best similarity metric for group fusion [18].

It is worth noting that despite the generally positive findings about the applicability of the Tanimoto coefficient, several of its weaknesses have also been reported from as early as in a 1998 study by Flower [19]. Around the same time, a tendency of the Tanimoto index to choose small compounds in dissimilarity selection was reported [20,21]. This finding was later corroborated and detailed by Holliday and coworkers [22]. Godden and coworkers reported the tendency of the Tanimoto index to produce similarity values around 1/3 even for structurally distant molecules [23].

In the literature (including several of the studies cited above) similarity measures are usually compared according to their performance in a few specific scenarios, such as the retrieval of molecules that are active on a specific protein, based on a limited number of reference compounds. Most of these studies (*e.g.* [13,16]) utilize databases of molecules that have previously been shown to be biologically relevant (*e.g.* MDDR or NCI anti-AIDS databases). In this paper we present a large-scale comparison of eight commonly available similarity metrics (Tanimoto, Dice, Cosine, Substructure [24] and Superstructure [25] similarities, and similarity definitions derived from the Manhattan, Euclidean and Soergel distances, see Equation 1) based on their rankings of the same datasets, using analysis of variance (ANOVA) and sum of ranking differences (SRD) [26,27]. Our goal was to study the ranking behavior of well-known and easily available similarity metrics on many independent datasets (modelling many independent scenarios of similarity searching), without any kind of *a priori* knowledge about the molecules involved. To that end, we have used a large supplier database (Mcule) of commercially available compounds for our calculations [28]. We also examine the effects of molecular size, selection method (*i.e*. random draw *vs*. deliberate selection of diverse molecules) and data pretreatment on the rankings and performances of the mentioned metrics.

## Methods

For the majority of the calculations, we have used KNIME [29], an open-source data analysis and cheminformatics software and the implementation of Chemaxon’s JChem [30] in KNIME. Molecules were drawn from the Mcule Purchasable Compounds Database (~5 M compounds) [28]. They were split into three categories based on their size: fragments, leadlike and druglike molecules (Table 1). An “All” category was also formed, where molecules were drawn regardless of size.

**Table 1:** **Size classes of molecules and their definitions**

**Class**	**Criteria**	**Total count in the Mcule database**	**Reference**
Fragment	M_W_ ≤ 250	166.458	[38]
	log*P* ≤ 3.5		
	rotB ≤ 5		
Leadlike	250 ≤ M_W_ ≤ 350	1.234.403	[39]
	log*P* ≤ 3.5		
	rotB ≤ 7		
Druglike	150 ≤ M_W_ ≤ 500	3.745.649	[40]
	log*P* ≤ 5		
	rotB ≤ 7		
	PSA < 150		
	HBD ≤ 5		
	HBA ≤ 10		

### Theory of similarity/distance measures

Most of the similarity and distance measures studied in this work are well-known and commonly used; their definitions are summarized in Table 2. Note that similarities and distances can be interconverted using the following equation [31]:

**Table 2:** **Formulas for the various similarity and distance metrics**

**Distance metric**	**Formula for continuous variables** ^**a**^	**Formula for dichotomous variables** ^**a**^
Manhattan distance	${D}_{A,\ B}={\displaystyle \sum_{j=1}^n}\left|{x}_{jA}-{x}_{jB}\right|$	*D*_*A*,*B*_ = *a* + *b* − 2*c*
Euclidean distance	$$ {D}_{A,\ B}={\left[{\displaystyle \sum_{j=1}^n}{\left({x}_{jA}-{x}_{jB}\right)}^2\right]}^{\raisebox{1ex}{$1$}\!\left/ \!\raisebox{-1ex}{$2$}\right.} $$	$$ {D}_{A,B}={\left[a+b-2c\right]}^{\raisebox{1ex}{$1$}\!\left/ \!\raisebox{-1ex}{$2$}\right.} $$
Cosine coefficient	$$ {S}_{A,B}=\left[{\displaystyle \sum_{j=1}^n}{x}_{jA}{x}_{jB}\right]/{\left[{\displaystyle \sum_{j=1}^n}{\left({x}_{jA}\right)}^2{\displaystyle \sum_{j=1}^n}{\left({x}_{jB}\right)}^2\right]}^{\raisebox{1ex}{$1$}\!\left/ \!\raisebox{-1ex}{$2$}\right.} $$	$$ {S}_{A,B}=\frac{c}{{\left[ ab\right]}^{\raisebox{1ex}{$1$}\!\left/ \!\raisebox{-1ex}{$2$}\right.}} $$
Dice coefficient	${S}_{A,B}=\left[2{\displaystyle \sum_{j=1}^n}{x}_{jA}{x}_{jB}\right]/\left[{\displaystyle \sum_{j=1}^n}{\left({x}_{jA}\right)}^2+{\displaystyle \sum_{j=1}^n}{\left({x}_{jB}\right)}^2\right]$	*S*_*A*,*B*_ = 2*c*/[*a* + *b*]
Tanimoto coefficient	${S}_{A,B}=\frac{\left[{\displaystyle {\sum}_{j=1}^n}{x}_{jA}{x}_{jB}\right]}{\left[{\displaystyle {\sum}_{j=1}^n}{\left({x}_{jA}\right)}^2+{\displaystyle {\sum}_{j=1}^n}{\left({x}_{jB}\right)}^2-{\displaystyle {\sum}_{j=1}^n}{x}_{jA}{x}_{jB}\right]}$	*S*_*A*,*B*_ = *c*/[*a* + *b* − *c*]
Soergel distance^b^	${D}_{A,\ B}=\left[{\displaystyle \sum_{j=1}^n}\left|{x}_{jA}-{x}_{jB}\right|\right]/\left[{\displaystyle \sum_{j=1}^n} max\left({x}_{jA},{x}_{jB}\right)\right]$	${D}_{A,B}=1-\frac{c}{\left[a+b-c\right]}$
Substructure similarity	See Ref [24]	
Superstructure similarity	See Ref [25]	

^a^S denotes similarities, while D denotes distances (according to the more commonly used formula for the given metric). Note that distances and similarities can be converted to one another using Equation **1**. *x*_*jA*_ means the *j*-th feature of molecule A. *a* is the number of *on* bits in molecule A, *b* is number of *on* bits in molecule B, while *c* is the number of bits that are *on* in both molecules.

^b^The Soergel distance is the complement of the Tanimoto coefficient.

1 $$ similarity=\frac{1}{1+ distance} $$

*i.e.* every similarity metric corresponds to a distance metric and *vice versa*. (From here on in this paper, we use the two definitions interchangeably). Since distances are always non-negative (R ∈ [0; + ∞]), similarity values calculated with this equation will always have a value between 0 and 1 (with 1 corresponding to identical objects, where the distance is 0). It is worth noting however, that the scales of different similarity metrics can be different, even though they cover the same range (*i.e.* 0 ≤ S ≤ 1). For example if the Euclidean distances of a group of objects from a reference object range from 5 to 8, their Euclidean similarities to the reference object will range from 1/9 to 1/6. Meanwhile, their Manhattan distances (which for dichotomous variables is equal to the Euclidean distances squared) will range from 25 to 64, meaning that their Manhattan similarities will range from 1/65 to 1/26.

A significant limiting factor in the selection of distance measures was that a large number of metrics are not defined for dichotomous variables. Thus, the mentioned six metrics were compared, with two graph-based similarity metrics (Substructure and Superstructure) implemented in JChem for KNIME in addition. These metrics are not defined in the same, purely mathematical manner as the other six, rather in an algorithmic approach, which is explained in detail in references [24,25] (Table 2).

Some metrics show highly similar behavior (identical in terms of ranking) with each other, which can be attributed to relationships in their definitions. For example, the Soergel distance is identical to the complement of the Tanimoto coefficient and both are monotonic with each other and with the Dice coefficient. The Manhattan and Euclidean coefficients are also monotonic. However, the relationships of these coefficients and their average are not linear. For example Dice *vs*. Average of Dice, Soergel, and Tanimoto coefficients provides a concave curve, while Soergel *vs*. Average is convex and Tanimoto *vs*. Average is slightly convex (see Additional file 1: Figure S8). Therefore, their average is a good option for data fusion. More detailed explanations are given by Willett in a 1998 article [32].

### Molecular fingerprints

A large number of methods exist to map molecular structures to bit strings (*i.e.* molecular fingerprints). Their classification, definitions and properties are covered in detail in the works of *e.g.* Bender and coworkers [5] or Cereto-Massagué and coworkers [7]. Based on the findings of Bender and coworkers (see Introduction), we first selected the ECFP4 fingerprint for our calculations.

However, a known characteristic of this fingerprint (and of the most dictionary-based fingerprints) is that it is quite sparse, *i.e.* relatively few bits are set to *on* (1). This results in a significant number of repeated similarity values in a dataset even as small as a hundred molecules. In ECFP4 fingerprints, at best one in every ten-twenty bits is *on*, meaning that there are on average 50–100 *on* bits in a 1024-bit fingerprint (see Additional file 1: Figure S1). As for two molecules (fingerprints), consider that 100 bit positions out of 1024 are “drawn” (set to *on*) twice: it can easily be seen that this can be carried out even without drawing a single common bit position, but extreme cases aside, the number of common *on* bits will likely take only a few possible values. Since the number of common *on* bits is present in the definition of every distance metric, the calculated similarity values will be degenerate as a result (here, “degenerations” mean repetitions: the same similarity values for different molecules). Unfortunately, this behavior cannot be influenced by adjusting either the diameter or the length of the fingerprint.

Since we did not want to impair the “resolution” of the similarity rankings, we were obliged to choose another type of fingerprint to study. (Another reason was a limitation of the SRD calculation in case of repeated observations (ties); namely at present the number of molecules/objects cannot exceed 40 [33]). Our next choice was the Chemaxon Chemical Fingerprint, a hashed fingerprint introduced in Chemaxon’s products, such as Jchem [34]. A significant advantage of this fingerprint over ECFPs is that it is “darker” (*i.e*. there are more *on* bits on average) and this “darkness” can even be tuned by adjusting a few parameters. The exchange of the studied fingerprint eliminated the mentioned problem almost completely.

### “Target” search

The term target has two meanings: drug targets such as pharmacologically relevant proteins; and target (reference) compounds in a similarity calculation. In this work, no protein targets were used; our goal was to reveal the ranking behavior of well-known and easily available similarity metrics on many independent datasets (modelling many independent scenarios of similarity searching), without any kind of *a priori* knowledge about the molecules involved. Hence active or inactive categories were not defined for the examined molecules. Have we taken one or a few specific scenarios of ligand-based virtual screening, we would have introduced some bias, as the relative performance of the metrics can vary with the reference compound. (See later Figure 3 and Additional file 1: Figure S7 as an example). Therefore, we have chosen to carry out a large number of experiments (1000) with randomly chosen reference compounds (and to statistically analyze the results). Due to the large number of experiments, the mentioned bias should be cancelled out to a large extent, if not entirely. In this work “target” is a reference compound that is randomly chosen for each of the 1000 runs. An sdf file with the target compounds of the similarity calculations (in the order of the SRD runs) is included as Additional file 2.

### Sum of ranking differences

**Figure 1:**
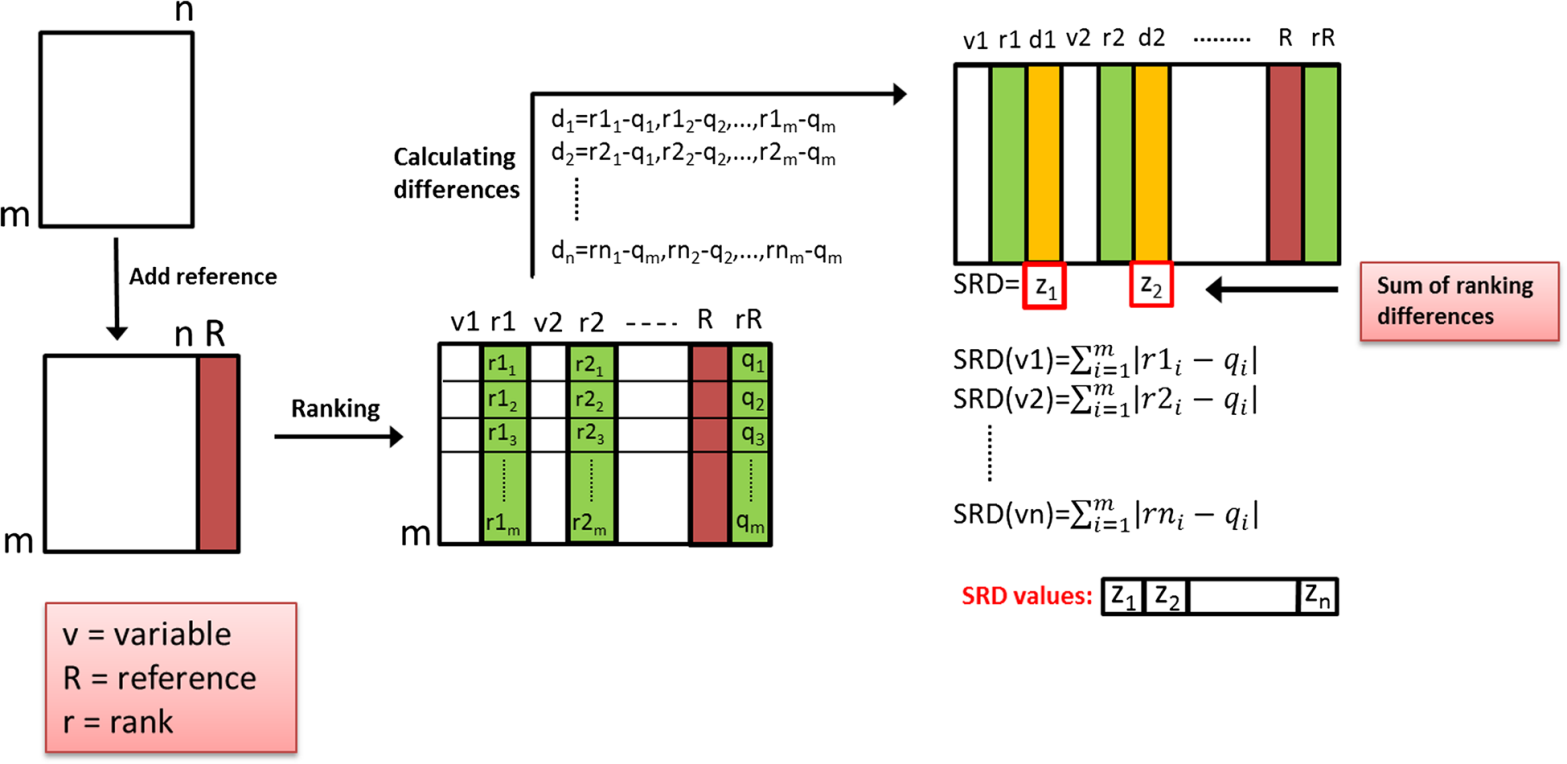
Scheme of the procedure to calculate sum of ranking differences. The input matrix contains similarity measures (n = 8) in the columns and molecules (m = 99) in the rows. A reference column (golden standard, here: average of the eight similarity measures) is added in the data fusion step (red). Then, all columns are doubled (green) and the molecules in each column are ranked by increasing magnitude (columns r1, r2, … rn). The differences (yellow columns) are calculated for each similarity measure and each molecule (*i.e.* each cell) between its rank (r1_1_, r1_2_ to rn_m_) and the rank assigned by the known reference method (rR = q_1_, q_2_, … q_m_). In the last step, the absolute values of the differences are summed up for each measure to give the final SRD values, which are to be compared. The smaller SRD means proximity to the reference, the smaller the better.

Sum of ranking differences is a novel and simple procedure [26,27,33] to compare methods, models, analytical techniques, *etc*. and it is entirely general. In the input matrix the objects (in the present case molecules) are arranged in the rows and the variables (models or methods, in the present case similarity measures) are arranged in the columns. The process of calculating the sum of ranking differences can be seen in Figure 1.

The input matrix contains similarity measures (*n* = 8) in the columns and molecules (*m* = 99) in the rows. A reference column (golden standard, benchmark) is added in the data fusion step (red). Then, all columns are doubled (green) and the molecules in each column are ranked by increasing magnitude (columns r1, r2, …, rn). The differences (yellow) between the ranks assigned by each similarity measure and by the known reference method (rR = q_1_, q_2_, …, q_m_) are computed for each object (molecule): *e.g.* for the first similarity measure: diff (r1_1_-q_1_), diff (r1_2_-q_2_), …, diff (r1_m_-q_m_). In the last step, the absolute values of the differences are summed up for each similarity measure to give the final SRD values. Such a way an SRD value is assigned to each similarity measure. (A summarizing animation of the SRD process is supplied as Additional file 3). Smaller SRD means proximity to the reference, the smaller the better. If the golden standard is not known, the average can be used for data fusion, which is the same as SUM fusion [17], because the number of columns (metrics) is the same for each row (molecule). The SRD procedure involves two validation steps. It is validated by a randomization test and a bootstrap-like cross-validation. Leave-one-out cross-validation is used if the number of objects is smaller than 14 whereas a seven-fold cross-validation is applied if the number of samples is higher than 13 [26].

## Results and discussion

### Input data generation

Our general objective in this study was to compare similarity metrics on a dataset as large as possible (and affordable). However, SRD has an intrinsic limitation regarding the number of objects: namely the calculation of the Gaussian random probability distribution curves becomes computationally intensive above sample sizes of 100–200 objects (the largest dataset processed in a reasonable amount of time so far is 1400 objects). For this reason, we have decided to split the dataset into smaller ones: a hundred molecules were drawn from the Mcule database for each SRD run (out of which one molecule was used as a reference), for a total of one thousand runs. Similarities were calculated between the remaining 99 molecules and the reference molecule, according to each similarity metric (those metrics that are originally defined as distances were converted to similarities according to Equation 1). The one thousand datasets were evenly distributed between the molecular size classes defined in the Methods section, as well as two selection methods: random draw *vs.* deliberately selecting diverse molecules (as implemented in the RDKit Diversity Picker tool in RDKit for KNIME [35]). An “All” size class was also defined: in this case molecules were drawn from the whole Mcule database, regardless to size. It was ensured that no molecules were ever drawn more than once. A summary of the prepared datasets is reported in Table 3.

**Table 3:** **Distribution of SRD runs in terms of molecule size and selection method**

**No. of SRD run**	**Size**	**Selection**	**Count**
0-124	Fragment	Random	125
125-249		Diverse	125
250-374	Leadlike	Random	125
375-499		Diverse	125
500-624	Druglike	Random	125
625-749		Diverse	125
750-874	All	Random	125
875-999		Diverse	125

### Statistical analysis

**Figure 2:**
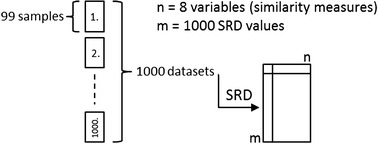
Scheme of the data generation. The SRD procedure was repeated 1000 times to eliminate the effect of random choices. Sum of ranking differences was calculated for 1000 data sets and gathered in an output file. The final output file contains a table with all of the SRD values for each similarity measure (n) on every dataset (m).

A specially developed sum of ranking differences routine (implemented in a Microsoft EXCEL VBA macro) was used for the evaluation of the dataset (1000*99 samples). Although the distances were converted into similarities (0–1), the measures still had different scales. Therefore, interval scaling (between 0 and 1) of the original values was applied as a data pretreatment method for the first time. The SRD macro generated an output file for each of the thousand datasets, which contained the scaled SRD values for every similarity measure. Another output file (SRDall) was generated at the same time, which contained a table with all of the SRD values for every dataset and similarity measure. The average was used as a “golden standard” in each SRD analysis. The reason for this choice follows from a simple assumption that all similarity measures express the true (unknown) similarities with some errors (biases and random errors, as well), so using the average, these errors are cancelled out at least partially. Using row-average can also be thought of as a consensus in accordance with the maximum likelihood principle, which “yields a choice of the estimator as the value for the parameter that makes the observed data most probable” [36]. Here, the average has the highest probability to happen in every case. For better understanding, Figure 2 presents the whole SRD process.

The steps above were repeated with standardization and rank transformation as data pretreatment methods. The scaling methods are given below:

2 $$ {x}_{i,j}\left( interval\  scaled\right)=\frac{x_{i,j}- min\left({x}_{i,j}\right)}{ \max \left({x}_{i,j}\right)- min\left({x}_{i,j}\right)} $$

3 $$ {x}_{i,j}(standardized)=\frac{x_{i,j}- average\left({x}_i\right)}{\mathrm{standard}\ \mathrm{deviation}\left({x}_i\right)} $$

Rank transformation has been carried out column-wise: *min*(*x*_*i*_) = 1. *max*(*x*_*i*_) = 99.

**Figure 3:**
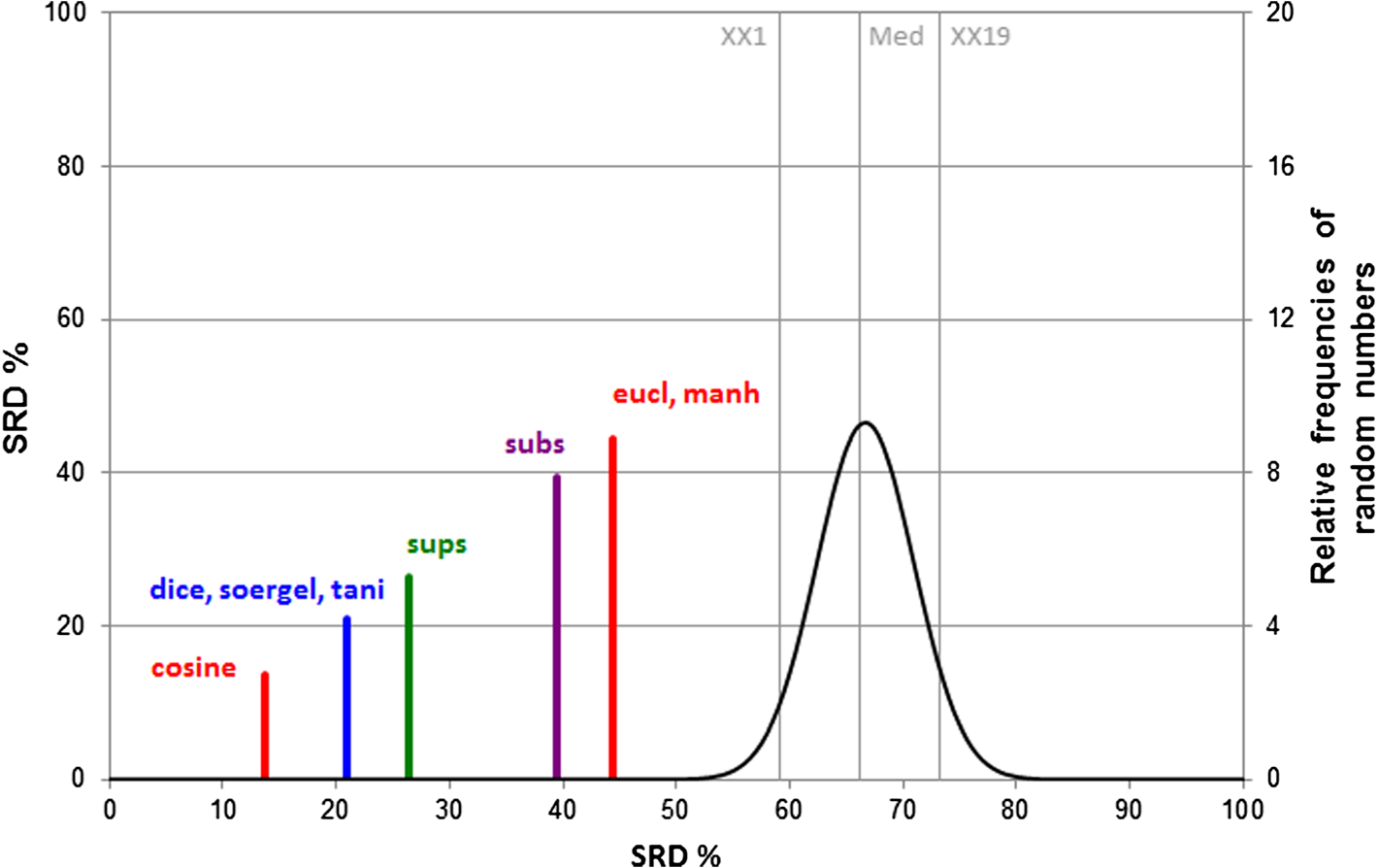
Visualization of SRD ranking and grouping. Average was used as reference. Scaled SRD values (between 0 and 100) are plotted on the *x* axis and left *y* axis. The right *y* axis shows the relative frequencies for the fitted Gauss curve on random numbers (black) (XX1 = 5% error limit, med = median, XX19 = 95% limit). If an SRD value (similarity metric) overlaps with the Gaussian curve, it is not distinguishable from random ranking.

SRD values are given on two scales. The first is the original one and the second is the scaled one between 0 and 100 denoted by SRD_nor_. On Figure 3 one of the thousand SRD results can be seen as an example. Here the scaled SRD values are used, which makes the models comparable. The equation of the scaling is:

4 $$ {\mathrm{SRD}}_{\mathrm{nor}}=100\mathrm{S}\mathrm{R}\mathrm{D}/{\mathrm{SRD}}_{\max }, $$

where SRD_max_ = the maximum attainable SRD value for the actual similarity measure.

Validation of the ranking has been carried out using a randomization test and a seven-fold cross-validation. For the former, a Gaussian random probability distribution curve is plotted, which helps us to decide whether the applied metric is better than or similar to the use of random ranks. For the latter, the dataset was split into seven subsets and then SRD values were calculated for each subset. SRDs calculated on the seven 6/7-th portions and the original SRD values define the uncertainty of the SRD values for each method. Without cross-validation, we would not know whether the colored lines on the diagram are indistinguishable or not (whether the distances between lines are negligible or statistically significant) .

For comparison an example is included in Additional file 1: Figure S7 that the ordering of similarity metrics is data set dependent. Figure S7 presents a dataset where the ranking of the similarity measures is quite different from the usual, *i.e*. Tanimoto and related metrics are not always the best based on SRD calculations. The large number of SRD calculations ensured that these random effects were accounted for and the space of possible reference compounds was thoroughly sampled. The distributions of the SRD values of the studied similarity metrics are included in the supplementary material (Additional file 1: Figure S5).

Each of the similarity measures is better than the use of random numbers (located outside the unacceptable region of the graph). The acceptable region is the first part of the plot, between zero and the line labeled XX1, which is the 5% error limit of the Gauss curve.

**Figure 4:**
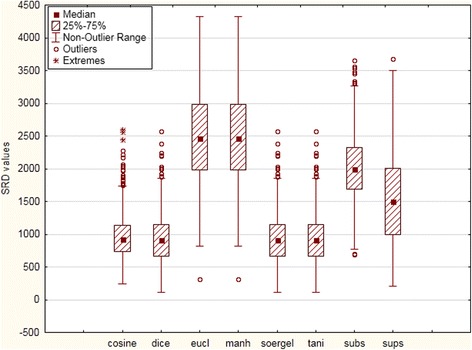
Box and whisker plot of the SRD values for eight similarity (and distance) metrics (with range scaling as data pretreatment method) in the SRDall dataset. The uncertainties (distribution) of SRD values reveal equivalent similarity metrics (e.g. Eucl and Manh). The high SRD values of the Euclidean, Manhattan and Substructure similarities indicate that their ranking behavior is significantly different from the average of the eight metrics (consensus), while Cosine, Dice, Soergel and Tanimoto similarities better represent the ranking based on the averages. The coefficient is 1 for non-outlier range. 1.5 coefficients is the limit for the outliers and over 1.5 coefficients the point is detected as an extreme value.

Box and whisker plots were made for the final dataset, which contained all SRD values for every dataset and similarity measure (SRDall). It clearly shows us the final result of the comparison. The plots were made for each of the three data pretreatment methods. Figure 4 shows the box and whisker plot of the SRDall dataset in the case of interval scaling as data pretreatment method. The box and whisker plots for the other two data pretreatment methods are included in Additional file 1: Figures S2 and S3.

The main conclusions from the box and whisker plots are that the Cosine, Dice, Tanimoto and Soergel similarity metrics are the most appropriate methods; they are the most reliable indices and stand closest to the average values (they have the smallest SRD values). Their equivalence follows from their definition and from the SRD procedure, as expected. Euclidean and Manhattan metrics have the largest median of SRD values on the box and whisker plots.

Since the Dice, Tanimoto and Soergel similarity metrics (and also, Manhattan and Euclidean) are closely related and have been shown here to produce identical rankings, one could argue that the reason they received the lowest SRD values is that their identical rankings weigh out the other metrics in the average values. To rule out this possibility, confirmatory calculations were undertaken. We have repeated the comparison for five metrics (omitting the Dice, Soergel and Manhattan similarities) to avoid the possibility of overweighting. The results gave the same ranking of the metrics; with only slight differences in the SRD values (see Additional file 1: Figure S4).

### Results of two-way ANOVA analysis

As SRD puts all influential factors on the same scale, a factorial ANOVA was applied to distinguish between the effects of factors. The effects of the following factors were investigated: (i) size classes, levels (4): fragment, leadlike, druglike, all, (ii) selection method of molecules, levels (2): random and diverse, (iii) scaling options (pretreatment methods), levels (3): interval scaling, standardization, rank transformation, and (iv) similarity indices, levels (8): Manhattan, Euclidean, Cosine, Dice, Tanimoto, Soergel, Substructure, Superstructure. All factors are significantly different (data not shown). For this case sum of ranking differences was used for every class separately. It means that the dataset – which included 1000 samples and eight variables (similarity metrics) – was built from parts, which contain 125 samples individually. (Table 3 clearly summarizes the distribution of SRD runs in terms of molecule size and selection method).

**Figure 5:**
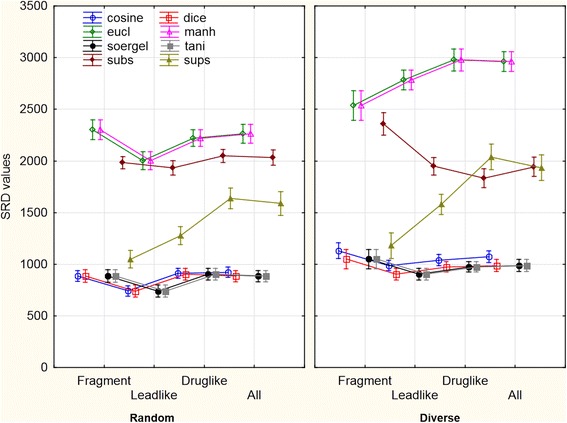
An illustrative example of two-way ANOVA (sigma restricted parametrization). A general, but not exclusive trend is to observe higher SRD values for the ranking of diversity picked molecules, which implies that the consensus of the discussed similarity metrics gets weaker as we investigate more diverse compound sets. Influential factors are shown using weighted means. The line plots are shifted on the categorical *x* axis horizontally for clarity. The vertical bars denote 0.95 confidence intervals.

Factorial ANOVA is a simple method to test the significance between average values of groups. For this purpose Statsoft STATISTICA 12.5 was applied [37]. The two factors included were the size (I1) and the selection method (I2). ANOVA analysis was carried out for datasets with different data pretreatment methods separately. For the interval scaled dataset, factorial ANOVA with sigma-restricted parameterization shows that both of the factors are significant; thus, the classes of the size and the selection method have large influence in the decision of the similarity metrics. The illustrative result of the test for interval scaled dataset is plotted on Figure 5.

For the Dice, Soergel and Tanimoto metrics, SRD values and their size dependence are identically equal (the small differences can be attributed to numerical uncertainties) and the same can be observed for the Euclidean and Manhattan similarity metrics. Substructure and Superstructure similarities have the largest variability for the examined molecules. While the best similarity metrics display virtually no size dependence, intriguing observations can be made about the other metrics. For example, Superstructure similarity tends to deviate more and more from the average at increasing molecular sizes. A similar trend can be observed for Euclidean/Manhattan, while the opposite holds for Substructure similarity, but only if the selection method is diversity picking.

Normal probability plots and normality tests were also carried out for the variables (it is reported in Additional file 1: Figure S5 and Table S1). Although the results show that the variables are not normally distributed, the very large dataset (one thousand samples) is sufficient in itself to carry out tests (factorial ANOVA), which require the assumption of normal distribution. Factorial ANOVA was carried out similarly to the standardized and rank scaled datasets, too. The two factors were also significant in every case, which supports the results of the factorial ANOVA for the interval scaled dataset. The plots were comparable to the results of the interval scaled matrix and no large differences were observed.

### Results of three-way ANOVA

Factorial ANOVA with three factors was also carried out. In this case the significance of different data pretreatment methods was also tested; it was the third factor for the ANOVA analysis. This version produced a more sophisticated picture than three one-way ANOVAs for the scaling methods separately, because here not just the significance was tested, but the interactions with the other factors (classes) as well. For this analysis sum of ranking differences was carried out for the entire dataset with different data pretreatment methods (3 × 1000 SRD runs).

**Figure 6:**
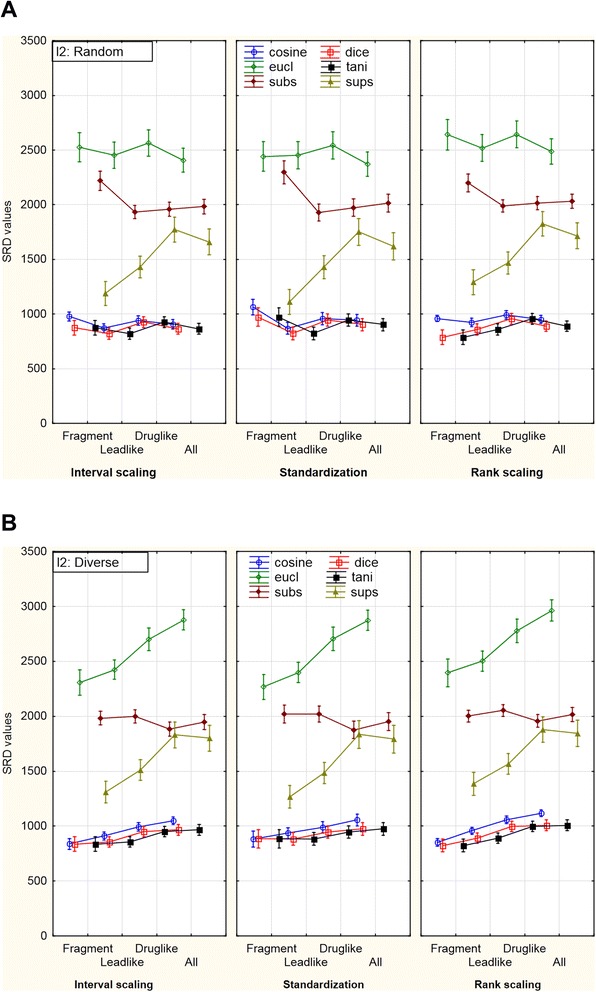
Effect of data pretreatment for the three-way ANOVA (sigma restricted parameterization). The changes of SRD values can be seen in different combinations of the factors. The data scaling methods are on the *x* axis and the selection method was: **(A)** *random draw*; **(B)** *diversity picking*. With random draw, Substructure similarities produce significantly higher SRD values for the ranking of fragment-like compounds than for bigger molecules. Meanwhile, with diversity picked molecules, Euclidean (and also Manhattan) similarities exhibit a trend to produce higher SRD values (*i.e.* deviate more from the consensus) as the size of the molecules increases. Weighted means were used for the creation of the plot. The vertical bars denote 0.95 confidence intervals. (Manhattan and Soergel similarities were omitted for clarity).

The result of factorial ANOVA with sigma-restricted parameterization showed that two interactions were not significant, namely the combination of the selection method and the data scaling method, and the combination of all of the three factors (see Additional file 1: Table S2). This latter case means that the factor of different data pretreatment methods is not significant in the combination of the other two factors. But it has to be noted that the factor of the different data pretreatment methods is significant alone. Figure 6A and B show the changes of SRD values in different combinations of the factors when the data scaling methods are on the *x* axis.

It is clearly shown that there are only little changes between the plots corresponding to the different data pretreatment methods. The SRD values are quite the same in every situation, which is reassuring. The shape of the lines is very similar, only a minor difference can be detected for the rank scaled results. The level of SRD values (except for Superstructure and Substructure) is somewhat higher mostly in the case of diverse selection. The Manhattan and the Soergel similarity metrics were omitted from the figure for clarity, because the results of the Tanimoto index is completely identical with that of the Soergel metric and the same holds for the Manhattan and the Euclidean metrics. Thus, the reason for the omission was solely the improvement of the visibility of the other distance metrics.

**Figure 7:**
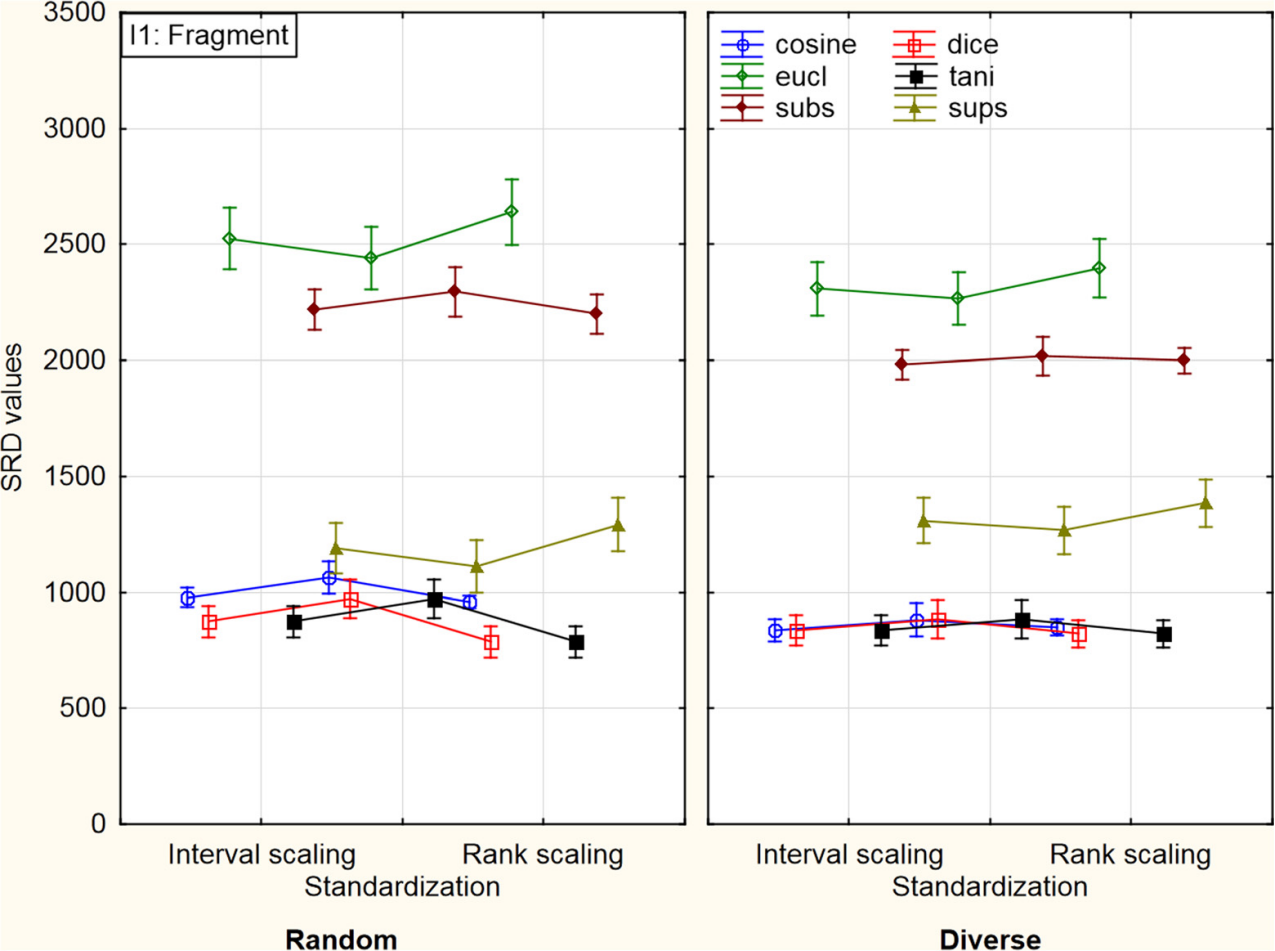
Comparison of diverse and random picking (three-way ANOVA with sigma restricted parameterization) in the case of fragment-like molecules. The SRD values in the case of standardization are quite different compared to the others. (This effect seems to be less pronounced for intentionally diverse molecules). Weighted means were used for the creation of the plot. The vertical bars denote 0.95 confidence intervals. (Manhattan and Soergel coefficients were omitted for clarity).

Another important result can be seen in Figure 7 where the factors were plotted in different arrangements; thus, a definite difference can be observed between the pattern in I1 factor’s first class (fragment) and the other three classes (plots for the other three classes are included in Additional file 1: Figures S6a, S6b and S6c).

The SRD values in the case of standardization are quite different compared to the others, whereas in the other two cases there is almost no difference in the average SRD values of the classes.

The 3000-sample dataset for the three-way ANOVA was prepared in two ways: (i) it was built from parts which contain 125 samples individually (same as for the two-way ANOVA) for each of the data pretreatment methods (125 × 8 × 3), and (ii) it was built from the entire datasets for each data pretreatment method (1000 × 3). It can also be concluded, that the results of the three-way ANOVA were not significantly different in these two cases (fragmented (125) SRD and entire SRD calculations).

## Conclusion

Statistical analysis of the ranking performances and correlations of eight similarity metrics was carried out with sum of ranking differences (SRD) and analysis of variance (ANOVA). Each similarity metric produced more reliable rankings than random numbers. Cosine, Dice, Tanimoto and Soergel similarities were identified as the best (equivalent) similarity metrics, while the similarity measures derived from Euclidean and Manhattan distances are far from being optimal. Nevertheless, this deviation from the other metrics makes them a good candidate for data fusion. It is important to note that in this context “best” means the metric that on its own produces the most similar rankings to those that the average of the studied eight metrics produces. In other words, the information content that is retrieved by taking all of the eight metrics into account is best represented by the mentioned four metrics. While this approach does not provide us information about the applicability of these similarity metrics in specific scenarios (such as identifying novel ligands for a given protein), it presents a much more general picture, where the metrics are compared to each other based on the results of a very large number of tasks (similarity calculations).

While our findings support previous observations about the Tanimoto coefficient and its equivalents [14,16], a more detailed and general picture is given regarding the rankings of the studied similarity metrics. We have shown that the Tanimoto-related (but not monotonic) Cosine coefficient is an equally appropriate choice.

Two-way ANOVA showed us that the factor of molecular size and the factor of selection method are significant separately and together as well in every case. It means that the results of the SRD analysis can be influenced by these two factors. Thus the outcome depends on the size of the molecules and the method of selection. In particular, the rankings of Euclidean, Manhattan, Substructure and Superstructure similarities have shown significant dependences on molecule size.

Although the factor of the different data pretreatment methods was significant at the 5% level, the significance depends on the evaluated similarity (or distance) metric/metrics. The difference between data pretreatment methods is barely observable.

We plan to extend the comparison for similarity metrics applied for non-dichotomous data and/or using SRD calculations in case of repeated items (degeneracies). Another possible extension of this study would involve the examination of less known similarity metrics.

## Additional files

Additional file 1:
**Supporting figures and tables.** Box and whisker plot of the SRD values for eight similarity and distance metrics (with standardization and rank scaling data pretreatment methods); box and whisker plot of the SRD values for five similarity and distance metrics; distribution of the SRD values of different similarity and distance metrics; results of the statistical tests for normal distribution; three-way ANOVA plots for “leadlike”, “druglike” and “all” molecular size classes (comparison of diverse and random picking); tests of significance for influential factors using three-way ANOVA; SRD example for a less frequent case; linear fits for three coefficients and their average values. Figures S1-S8 and Tables S1, S2. Additional file 2:
**Target compounds of the similarity calculations (1000).**
Additional file 3:
**A simple animation to illustrate how SRD works.**

## Abbreviations

eucl: Euclidean distance
HBA/HBD: Number of hydrogen-bond acceptors/donors
log*P*: Logarithm of the *n*-octanol/water partition coefficient
manh: Manhattan distance
MW: Molecular weight
PSA: Polar surface area
rotB: Number of rotatable bonds
SRD: Sum of ranking differences
subs: Substructure similarity
sups: Superstructure similarity
tani: Tanimoto index

## References

[1] Bender A, Glen RC (2004). Molecular similarity: a key technique in molecular informatics. Org Biomol Chem.

[2] Maggiora G, Vogt M, Stumpfe D, Bajorath J (2014). Molecular similarity in medicinal chemistry. J Med Chem.

[3] Kubinyi H (1998). Similarity and dissimilarity: a medicinal chemist’s view. Perspect Drug Discov Des.

[4] Eckert H, Bajorath J (2007). Molecular similarity analysis in virtual screening: foundations, limitations and novel approaches. Drug Discov Today.

[5] Bender A, Jenkins JL, Scheiber J, Sukuru SCK, Glick M, Davies JW (2009). How similar are similarity searching methods?: a principal component analysis of molecular descriptor space. J Chem Inf Model.

[6] Rogers D, Hahn M (2010). Extended-connectivity fingerprints. J Chem Inf Model.

[7] Cereto-Massagué A, Ojeda MJ, Valls C, Mulero M, Garcia-Vallvé S, Pujadas G (2015). Molecular fingerprint similarity search in virtual screening. Methods.

[8] Kokare M, Chatterji BN, Biswas PK, Edited by IEEE (2003). Comparison of similarity metrics for texture image retrieval. Proceedings of TENCON 2003 Conference on Convergent Technologies for the Asia-Pacific Region.

[9] Strehl A, Strehl E, Ghosh J, Mooney R, Edited by AAAI (2000). Impact of similarity measures on web-page clustering. Proceedings of the Workshop on Artificial Intelligence for Web Search (AAAI 2000).

[10] Becker H, Naaman M, Gravano L (2010). Learning similarity metrics for event identification in social media. Proceedings of the third ACM international conference on Web search and data mining.

[11] Reisen F, Zhang X, Gabriel D, Selzer P (2013). Benchmarking of multivariate similarity measures for high-content screening fingerprints in phenotypic drug discovery. J Biomol Screen.

[12] Holliday JD, Hu C-Y, Willett P (2002). Grouping of coefficients for the calculation of inter-molecular similarity and dissimilarity using 2D fragment Bit-strings. Comb Chem High Throughput Screen.

[13] Chen X, Reynolds CH (2002). Performance of similarity measures in 2D fragment-based similarity searching: comparison of structural descriptors and similarity coefficients. J Chem Inf Comput Sci.

[14] Salim N, Holliday J, Willett P (2003). Combination of fingerprint-based similarity coefficients using data fusion. J Chem Inf Comput Sci.

[15] Willett P (2006). Similarity-based virtual screening using 2D fingerprints. Drug Discov Today.

[16] Todeschini R, Consonni V, Xiang H, Holliday J, Buscema M, Willett P (2012). Similarity coefficients for binary chemoinformatics data: overview and extended comparison using simulated and real data sets. J Chem Inf Model.

[17] Willett P (2013). Combination of similarity rankings using data fusion. J Chem Inf Model.

[18] Whittle M, Gillet VJ, Willett P, Alex A, Loesel J (2004). Enhancing the effectiveness of virtual screening by fusing nearest neighbor lists: a comparison of similarity coefficients. J Chem Inf Comput Sci.

[19] Flower DR (1998). On the properties of Bit string-based measures of chemical similarity. J Chem Inf Comput Sci.

[20] Lajiness MS (1997). Dissimilarity-based compound selection techniques. Perspect Drug Discov Des.

[21] Dixon SL, Koehler RT (1999). The hidden component of size in two-dimensional fragment descriptors: side effects on sampling in bioactive libraries. J Med Chem.

[22] Holliday JD, Salim N, Whittle M, Willett P (2003). Analysis and display of the size dependence of chemical similarity coefficients. J Chem Inf Comput Sci.

[23] Godden JW, Xue L, Bajorath J (2000). Combinatorial preferences affect molecular similarity/diversity calculations using binary fingerprints and Tanimoto coefficients. J Chem Inf Comput Sci.

[24] Yan X, Yu P, Han J, Edited by ACM (2005). Substructure similarity search in graph databases. Proceedings of the 2005 ACM SIGMOD international conference on Management of data.

[25] Klinger S, Austin J (2006). Weighted superstructures for chemical similarity searching. Proceedings of the 9th Joint Conference on Information Sciences.

[26] Héberger K, Kollár-Hunek K (2011). Sum of ranking differences for method discrimination and its validation: comparison of ranks with random numbers. J Chemom.

[27] Héberger K (2010). Sum of ranking differences compares methods or models fairly. TrAC Trends Anal Chem.

[28] Kiss R, Sándor M, Szalai FA (2012). http://Mcule.com: a public web service for drug discovery. J Cheminform.

[29] KNIME | Konstanz Information Miner, University of Konstanz, Germany. 2014. [https://www.knime.org/]

[30] JChem 2.8.2, ChemAxon LLC, Budapest, Hungary. 2014 [http://www.chemaxon.com]

[31] Todeschini R, Ballabio D, Consonni V, Mauri A, Pavan M (2007). CAIMAN (classification and influence matrix analysis): a new approach to the classification based on leverage-scaled functions. Chemom Intell Lab Syst.

[32] Willett P, Barnard J, Downs G (1998). Chemical similarity searching. J Chem Inf Comput Sci.

[33] Kollár-Hunek K, Héberger K (2013). Method and model comparison by sum of ranking differences in cases of repeated observations (ties). Chemom Intell Lab Syst.

[34] Chemical Hashed Fingerprint [https://docs.chemaxon.com/display/CD/Chemical+Hashed+Fingerprint].

[35] RDKit: Cheminformatics and Machine Learning Software, Open-source. 2014. [http://www.rdkit.org/]

[36] Hastie T, Tibshirani R, Friedman J (2001). Overview of supervised learning. Elements of Statistical Learning: Data Mining, Inference, and Prediction.

[37] STATISTICA 12.5, StatSoft, Inc., Tulsa, OK 74104, USA, 2014. [http://www.statsoft.com/Products/STATISTICA-Features/Version-12].

[38] Carr RAE, Congreve M, Murray CW, Rees DC (2005). Fragment-based lead discovery: leads by design. Drug Discov Today.

[39] Teague SJ, Davis AM, Leeson PD, Oprea T (1999). The design of leadlike combinatorial libraries. Angew Chemie Int Ed.

[40] Lipinski CA (2000). Drug-like properties and the causes of poor solubility and poor permeability. J Pharmacol Toxicol Methods.

