# Supplementary material for: Why is Tanimoto index an appropriate choice for fingerprint-based similarity calculations?
Source: J Cheminform. 2015 May 20;7:20. doi: 10.1186/s13321-015-0069-3 (PMC4456712; doi:10.1186/s13321-015-0069-3)
Supplement: Additional file 1: — Supporting figures and tables. Box and whisker plot of the SRD values for eight similarity and distance metrics (with standardization and rank scaling data pretreatment methods); box and whisker plot of the SRD values for five similarity and distance metrics; distribution of the SRD values of different similarity and distance metrics; results of the statistical tests for normal distribution; three-way ANOVA plots for “leadlike”, “druglike” and “all” molecular size classes (comparison of diverse and random picking); tests of significance for influential factors using three-way ANOVA; SRD example for a less frequent case; linear fits for three coefficients and their average values. Figures S1-S8 and Tables S1, S2. [file 13321_2015_69_MOESM1_ESM.docx]

**Supplementary materials**

**Figure S1:** Snapshot from KNIME. ECFP fingerprints are scarce in “on” bits, but “on” bits are more frequent in Chemaxon Chemical Fingerprints. (To put it another way, Chemical Fingerprints are “darker” than ECFP fingerprints.) The rareness of “on” bits in ECFP fingerprints gives rise to degenerate similarity values when comparing large numbers of molecules using this fingerprint.


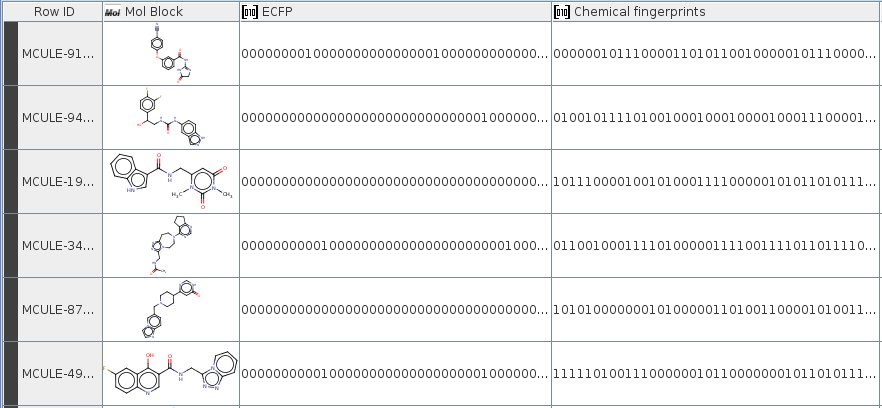


**Figure S2:** Box and whisker plot of the SRD values for eight similarity and distance metrics (with **standardization** data pretreatment method) in the SRDall dataset. The coefficient is 1 for non-outlier range. 1.5 coefficient is the limit for the outliers and over 1.5 coefficient the point is detected as extreme value.


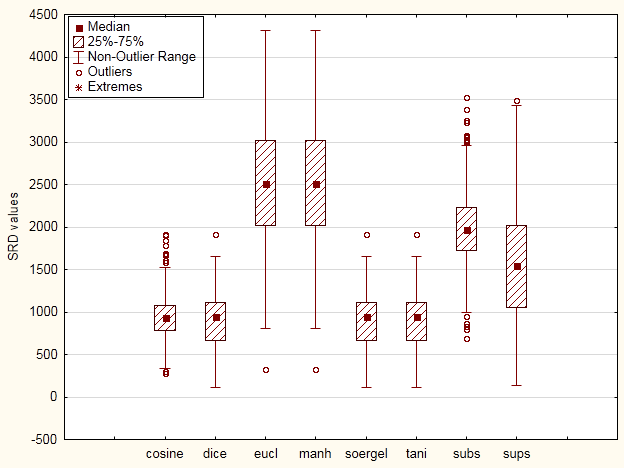


**Figure S3:** Box and whisker plot of the SRD values for eight similarity and distance metrics (with **rank scaling** as data pretreatment method) in the SRDall dataset. The coefficient is 1 for non-outlier range. 1.5 coefficient is the limit for the outliers and over 1.5 coefficient the point is detected as extreme value.


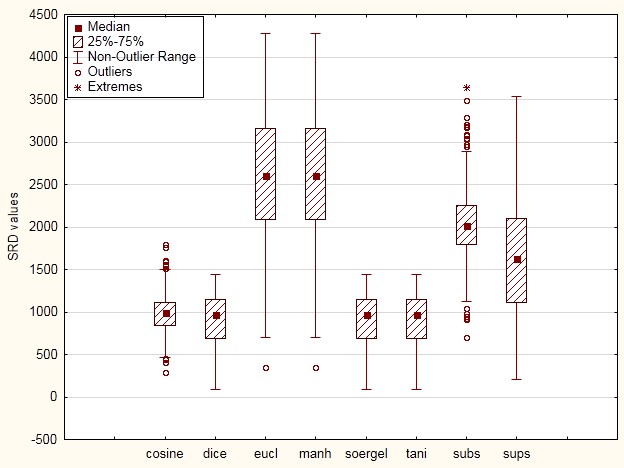


**Figure S4:** Box and whisker plot of the SRD values for five similarity and distance metrics (with **interval scaling** data pretreatment method) in the SRDall dataset (confirmatory calculation). The coefficient is 1 for non-outlier range. 1.5 coefficient is the limit for the outliers and over 1.5 coefficient the point is detected as extreme value.


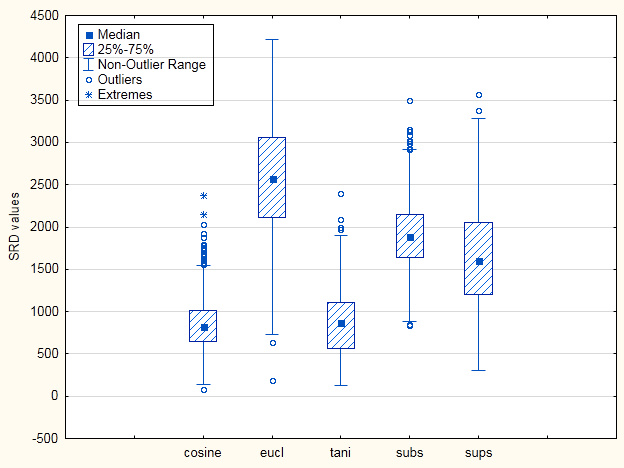


**Figure S5:** The deviations from normal distributions of the SRD values of different similarity and distance metrics (SRDall dataset was used with interval scaling as data pretreatment method).


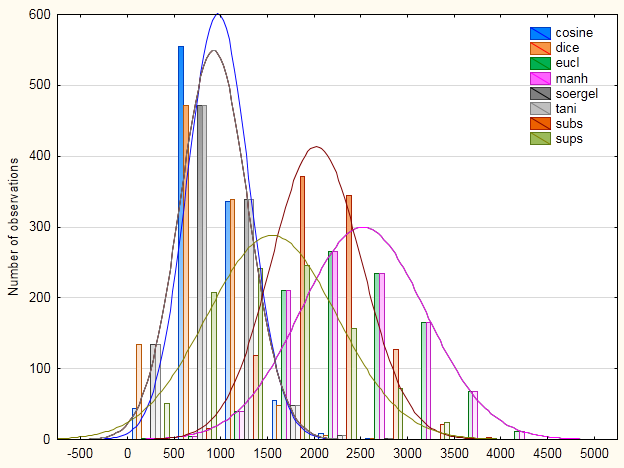


**Figure S6a:** Comparison of diverse and random picking (three-way ANOVA with sigma restricted parameterization) in the case of leadlike molecular size. Weighted means were used for the creation of the plot. The vertical bars denote 0.95 confidence intervals.

**
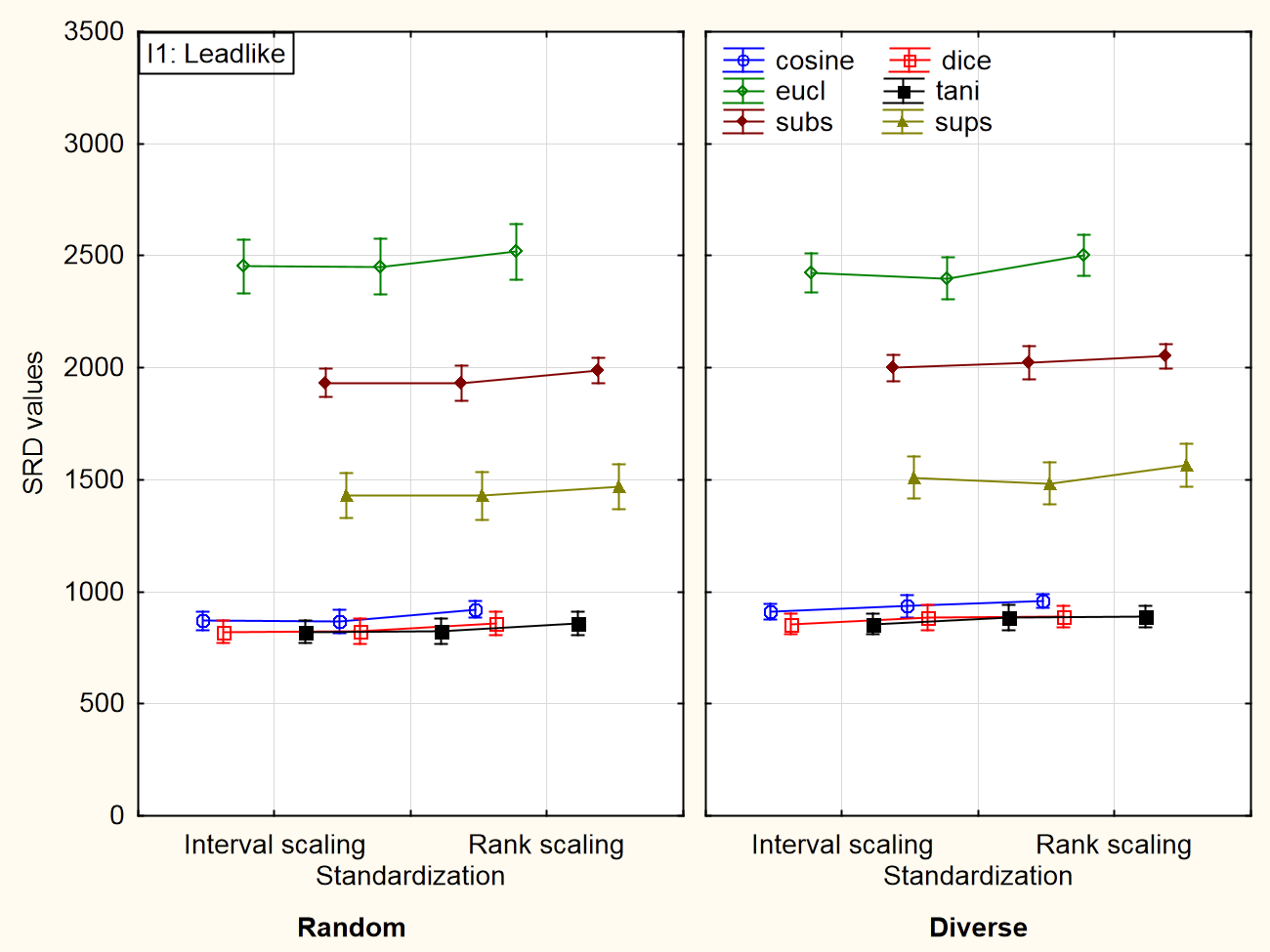
**

**Figure S6b:** Comparison of diverse and random picking (three-way ANOVA with sigma restricted parameterization) in the case of druglike molecular size. Weighted means were used for the creation of the plot. The vertical bars denote 0.95 confidence intervals.


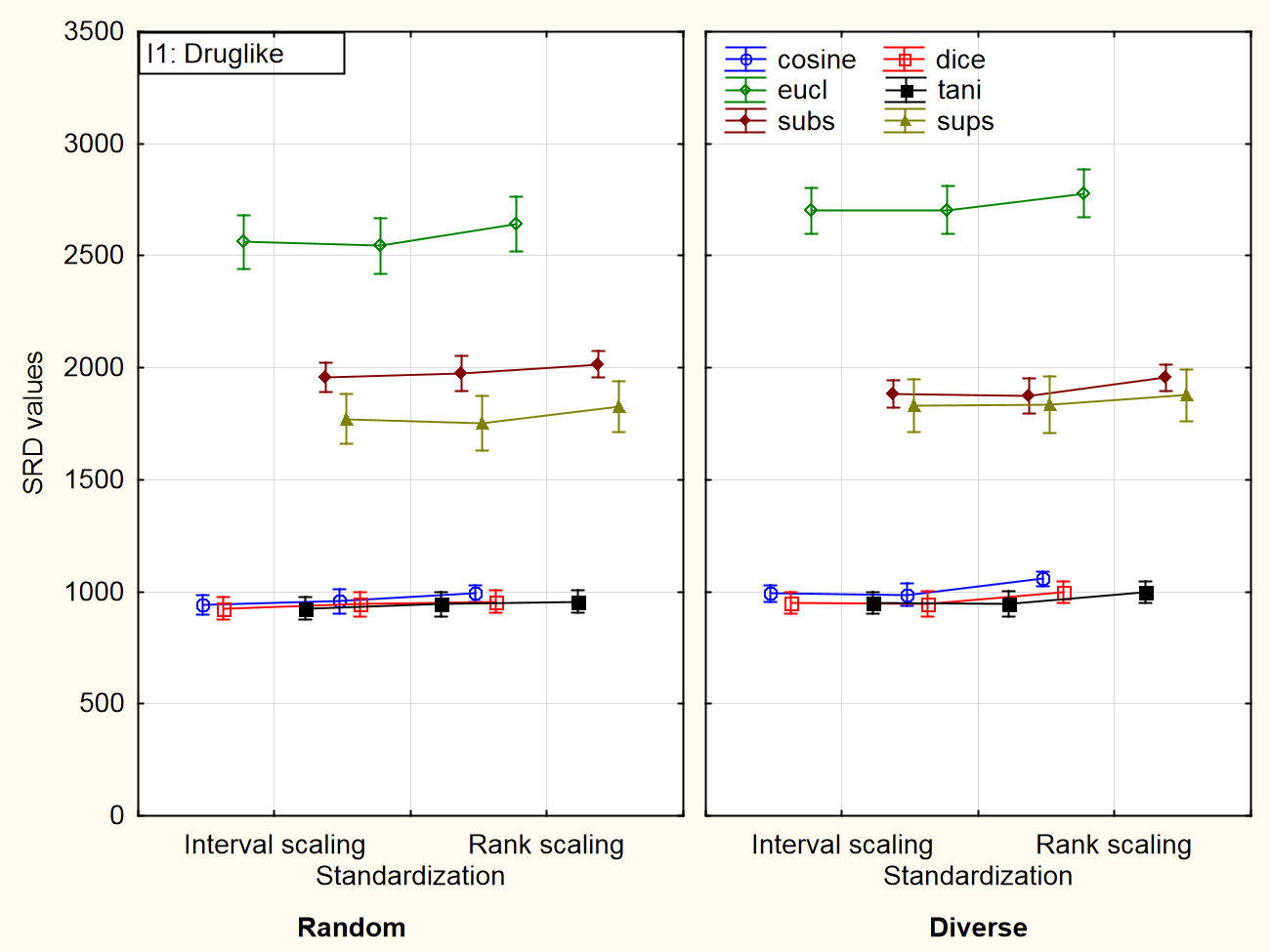


**Figure S6c:** Comparison of diverse and random picking (three-way ANOVA with sigma restricted parameterization) in the case of all molecular size. Weighted means were used for the creation of the plot. The vertical bars denote 0.95 confidence intervals.


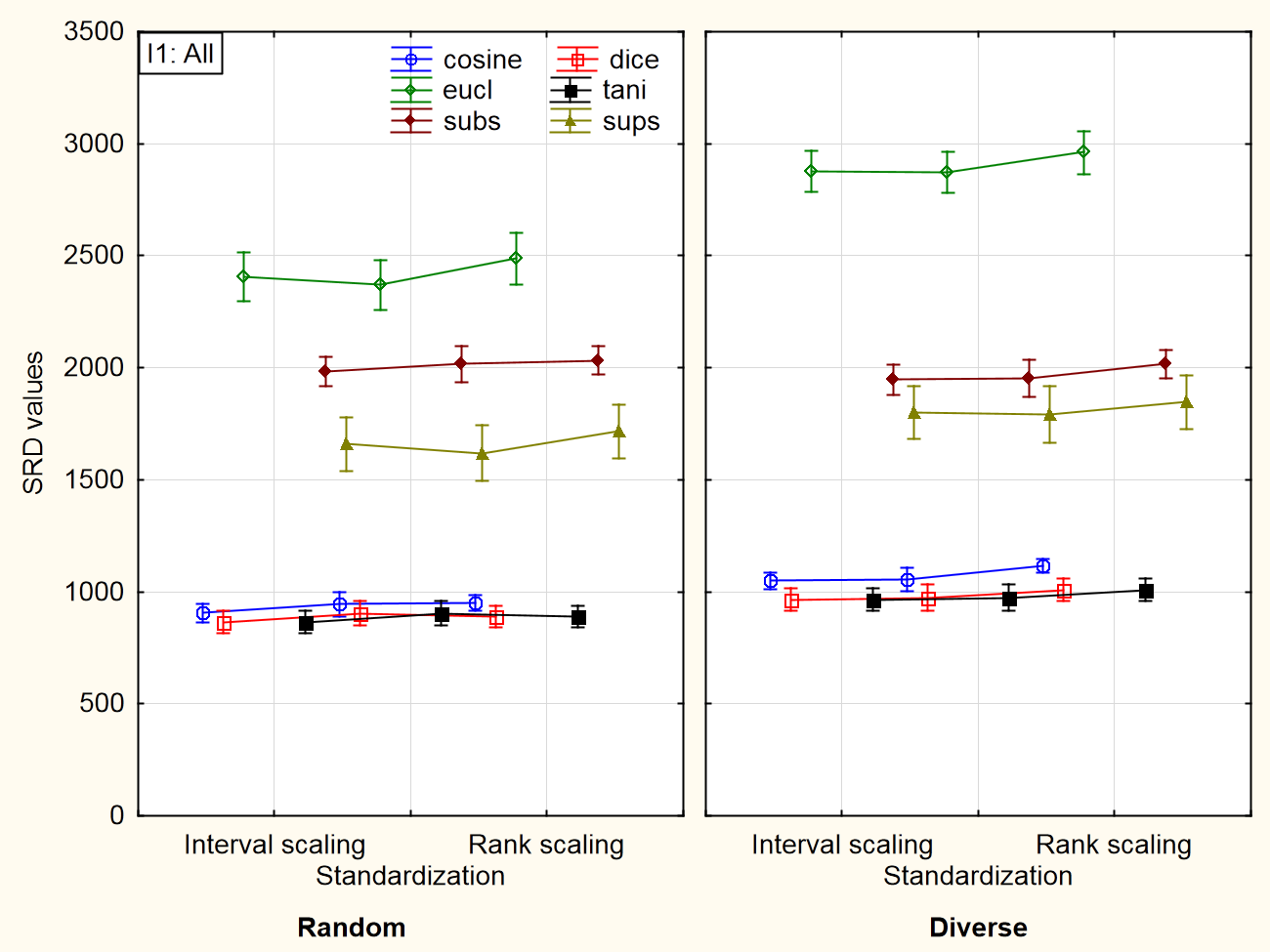


**Figure S7:** For comparison an example is shown that the ordering of similarity metrics is data set dependent. Average was used as reference. Scaled SRD values (between 0 and 100) are plotted on the *x* axis and left *y* axis. The right *y* axis shows the relative frequencies for the black (fitted) Gauss curve (XX1= 5 % limit, med= median, XX19= 95 % limit).

**
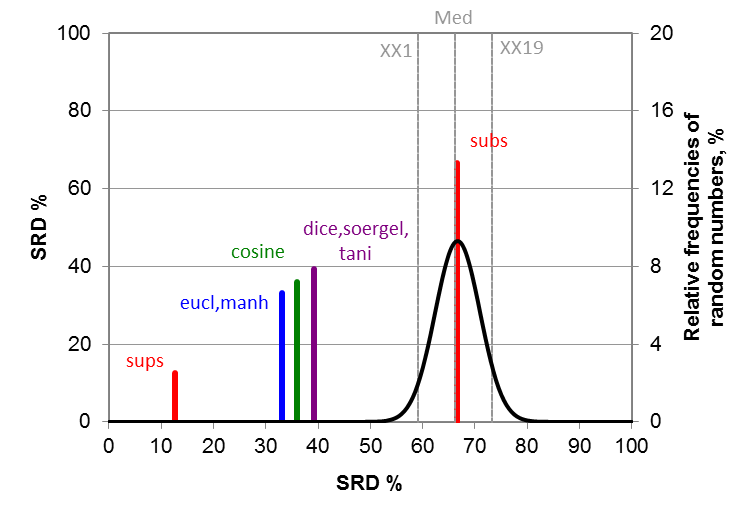
**

**Figure S8:** Linear fit of different coefficients vs. the average of the three coefficients. The linear fits are marked by the red line and their confidence bands (95 %) are marked by the red dashed lines. Dice *vs*. Average of Dice, Soergel and Tanimoto coefficients provides a concave curve (a), while Soergel *vs*. Average is convex (b) and Tanimoto *vs*. Average is slightly convex (c).

**
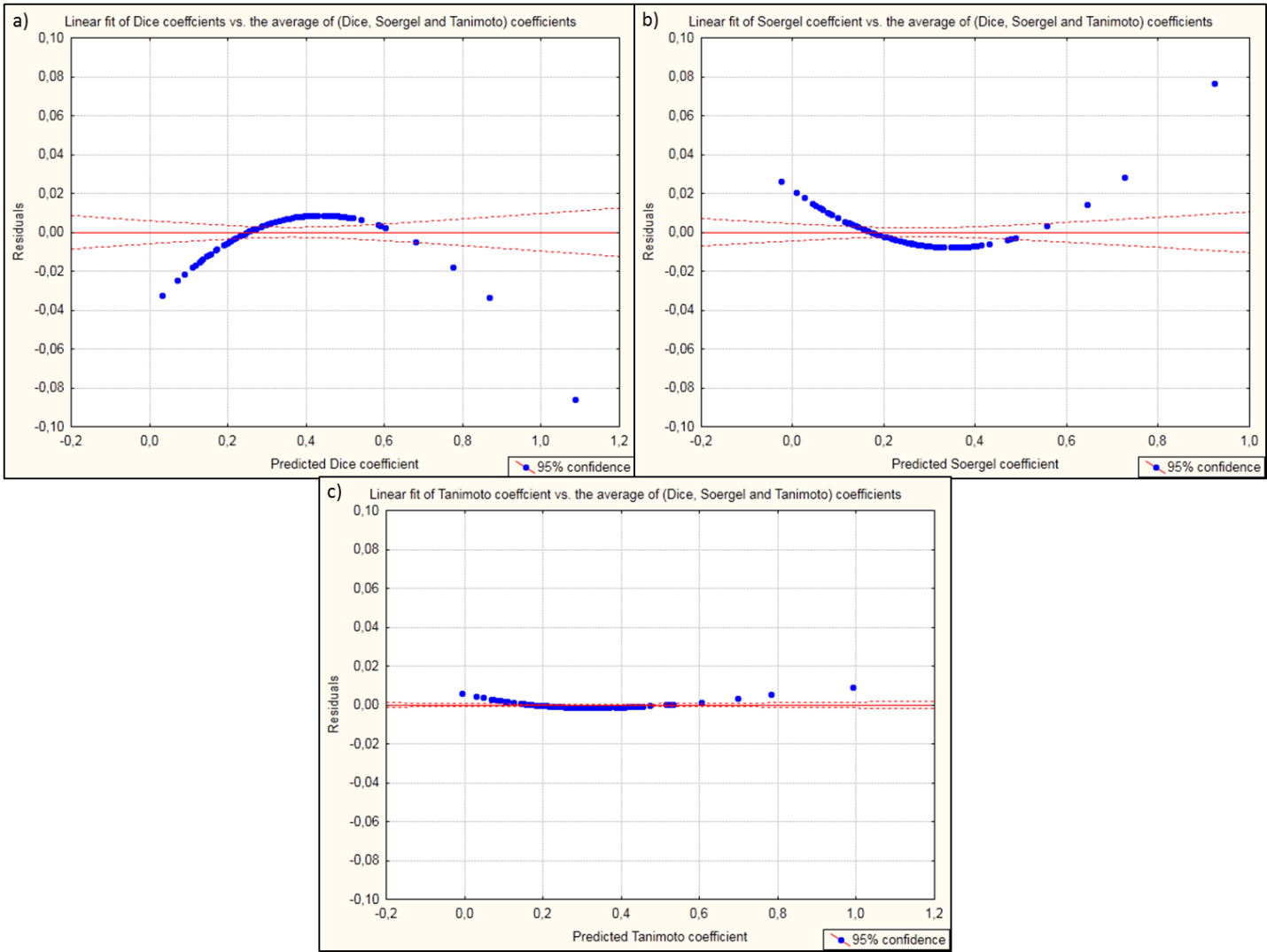
**

**Table S1:** *p* Values of the statistical tests for normal distribution. (the null hypothesis can be rejected below p=0.05)

| Used test | Cosine | Dice | Eucl | Manh | Soergel | Tani | Subs | Sups |
| --- | --- | --- | --- | --- | --- | --- | --- | --- |
| Kolmogorov-Smirnov | <0.01 | >0.20 | <0.05 | <0.05 | >0.20 | >0.20 | >0.20 | <0.1 |
| Lilliefors | <0.01 | <0.01 | <0.01 | <0.01 | <0.01 | <0.01 | <0.05 | <0.01 |
| Shapiro-Wilk’s | 0.000 | 0.000 | 0.000 | 0.000 | 0.000 | 0.000 | 0.07163 | 0.000 |

**Table S2:** Tests of significance for influential factors using three-way ANOVA (sigma-restricted parameterization and effective hypothesis decomposition). Significant factors and factor combinations are bold.

Effect Test Value F Effect Error p

df df

Intercept **Wilks 0.01110258 44104.0937 6 2971 0.0000**

I1 **Wilks 0.78137491 42.5502734 18 8403.74 0.0000**

12 **Wilks 0.97595606 12.1990712 6 2971.00 0.0000**

I3 **Wilks 0.97213127 7.04748345 12 5942.00 0.0000**

I1*12 **Wilks 0.91102150 15.6384215 18 8403.74 0.0000**

I1*I3 **Wilks 0.98026741 1.64885499 36 13049.34 0.0086**

12*I3 Wilks 0.99750659 0.618483741 12 5942.00 0.8284

I1*12*I3 Wilks 3.356131E+15 36 13049.34
