# Supplementary figures and images for: Why is Tanimoto index an appropriate choice for fingerprint-based similarity calculations?
Source: J Cheminform. 2015 May 20;7:20. doi: 10.1186/s13321-015-0069-3 (PMC4456712; doi:10.1186/s13321-015-0069-3)

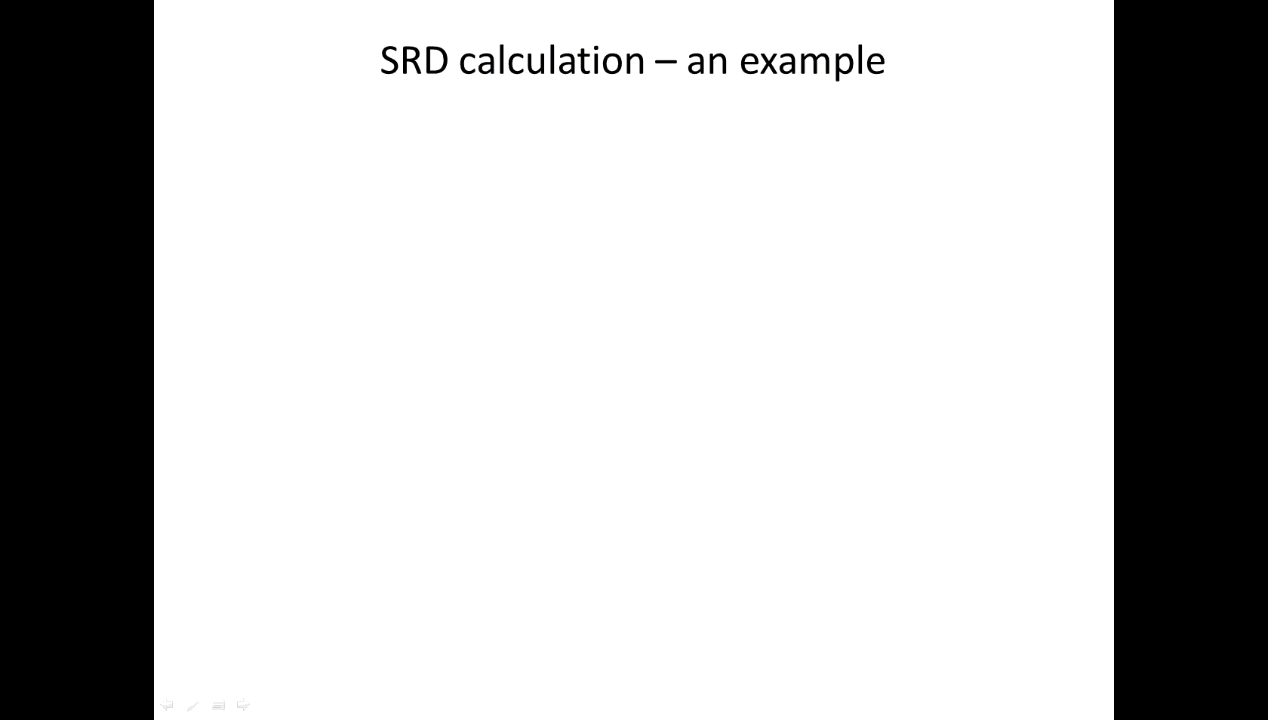

Supplement: Additional file 3: — A simple animation to illustrate how SRD works. [file 13321_2015_69_MOESM3_ESM.gif]
